# JNK3 as Therapeutic Target and Biomarker in Neurodegenerative and Neurodevelopmental Brain Diseases

**DOI:** 10.3390/cells9102190

**Published:** 2020-09-28

**Authors:** Clara Alice Musi, Graziella Agrò, Francesco Santarella, Erika Iervasi, Tiziana Borsello

**Affiliations:** 1 Department of Pharmacological and Biomolecular Sciences, Milan University, 20133 Milan, Italy; claraalice.musi@marionegri.it; 2 Department of Neuroscience, Istituto di Ricerche Farmacologiche Mario Negri-IRCCS, 20156 Milan, Italy; graziella.agro@guest.marionegri.it (G.A.); francesco.santarella@marionegri.it (F.S.); erika.iervasi@libero.it (E.I.); 3 Department of Experimental Medicine, University of Genoa, Via De Toni 14, 16132 Genoa, Italy

**Keywords:** JNK, synaptic dysfunction, JIP-1, β-arrestin-2, Alzheimer’s disease, neuroprotection

## Abstract

The c-Jun *N*-terminal kinase 3 (JNK3) is the JNK isoform mainly expressed in the brain. It is the most responsive to many stress stimuli in the central nervous system from ischemia to Aβ oligomers toxicity. JNK3 activity is spatial and temporal organized by its scaffold protein, in particular JIP-1 and β-arrestin-2, which play a crucial role in regulating different cellular functions in different cellular districts. Extensive evidence has highlighted the possibility of exploiting these adaptors to interfere with JNK3 signaling in order to block its action. JNK plays a key role in the first neurodegenerative event, the perturbation of physiological synapse structure and function, known as synaptic dysfunction. Importantly, this is a common mechanism in many different brain pathologies. Synaptic dysfunction and spine loss have been reported to be pharmacologically reversible, opening new therapeutic directions in brain diseases. Being JNK3-detectable at the peripheral level, it could be used as a disease biomarker with the ultimate aim of allowing an early diagnosis of neurodegenerative and neurodevelopment diseases in a still prodromal phase.

## 1. The MAPK Family

The exposure to extra- or intracellular stress disrupts cellular homeostasis and induces a cell-response mediated by the mitogen-activated protein kinases (MAPKs) signal transduction pathway. MAPKs are subdivided into three major groups:

1. Extracellular signal regulated kinase (ERK);

2. Mitogen activated protein kinase p38 (p38);

3. c-Jun NH_2_-terminal kinase (JNK) [1].

**Figure 1:**
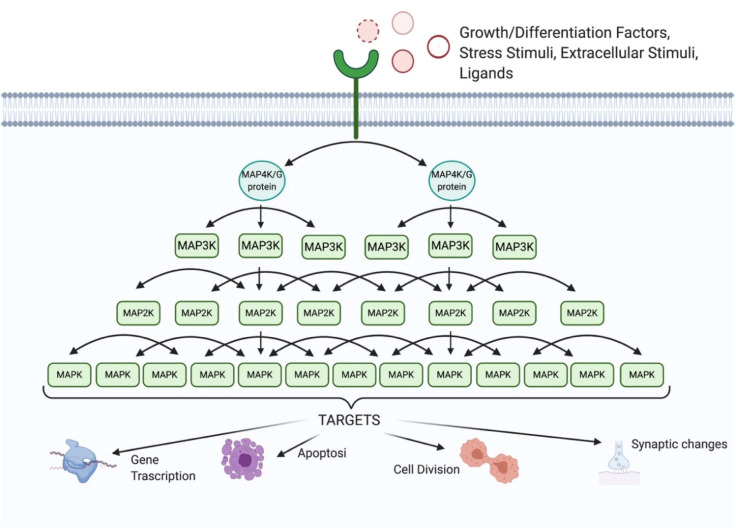
Signal amplification of the MAPKs cascade. Signal transduction occurs by a phosphorylation mechanism: three different MAPKs activate each other, by double phosphorylation, amplifying the signal. The first members of this signaling-module are MAPKK kinases (MAP3K), which are activated in response to extracellular stimuli; once activated, they phosphorylate a downstream MAPK kinase (MAP2K), which in turn activates a MAPK. The interaction between upstream regulators and MKP (MAPK phosphatases) determines the MAPK-activation status.

Each MAPK is activated by dual phosphorylation; this phosphorylation is mediated by a MAPK kinase (MAP2K) that is activated by the phosphorylation of another MAPKK kinase (MAP3K). MAPKs are able to amplify the respond to stimuli inside the cell through the kinase signaling cascade (Figure 1) acting on different cellular substrates, proteins and factors.

MAP3Ks are serine/threonine protein kinases that represent the first members of the mitogen-activated protein kinase (MAPK) signaling module [2]. Over the years, at least twenty MAP3Ks have been identified in mammals. The most widely-studied ones are the well characterized ASK1 (apoptosis signal regulating kinase 1), TAK1 (transforming-growth-factor-β-activated kinase 1), MEKK1–4 (MAPK/extracellular-signal-regulated kinase kinase kinase 1–4) and MLK3 (mixed-lineage kinase 3) [2].

The specific transmission of the signals from the upstream kinase (MAP3Ks) to the next kinase (MAP2Ks) is ensured by direct protein–protein interactions between the components of the cascade or through scaffold proteins [2].

MAP3Ks have been considered the gate keepers of MAPK pathways; in fact, many MAP3K are subject to self-inhibition (for example MEKK1, MEKK4, MLK3) thanks to various regulatory domains associated with the kinase domains [3,4].

Complete activation of MAP3K also requires self-phosphorylation through a transient tetrameric complex that increases MAP3K stability and efficacy [5,6]. Other auxiliary kinases, generally classified as MAP4K, can mediate these and other MAP3K modifications.

The second member of the MAP module is MAPK kinase-kinase (MAP2K), which activates the effector of this pathway (MAP kinase). Currently, there are seven different MAP2Ks identified in mammalian cells. The different MAP2K isoforms show substrate selectivity towards MAPK family members.

For example, the MAP2Ks are known as MKK3 and MKK6 that phosphorylate p38; MKK7 phosphorylates JNK, while MKK4 can phosphorylate both substrates. Instead, MKK1 and MKK2 are the MAP2Ks for ERK1/2 [7].

Importantly, MKK4 and MKK7 are the only two upstream activators of JNK [8]. Under physiological conditions, the two kinases act in a synergistic manner in order to double-phosphorylate JNKs [9].

The MAPKs cascade mediates the response to several stimuli that influence cellular activity and cell survival. MAPKs are involved in the transmission of external stimuli inside the cell, amplifying the signal, and by this regulating cellular functions, including proliferation, gene expression, differentiation, mitosis, cell survival and apoptosis. The MAPK cascade not only amplifies the initial message and enhances sensitivity of the whole system, generating a switch-like response [10], but it also promotes proofreading. Furthermore, the cascade structure in MAPK attenuates the crosstalk among MAPK and the noise [11], thus increasing the specificity of the molecular pathway [12].

The extent and timing of MAPK activation are also controlled by MAPK phosphatases (MKPs), which are highly conserved enzymes involved in the regulation of MAPK signaling. Unique to eukaryotes, MKPs inactivate MAPKs by removing phosphate groups from specific phosphorylated amino-acid residues in the MAPKs’ activation site. MKPs are classified into three subgroups: tyrosine-specific phosphatases, serine-threonine phosphatases or dual-specificity (threonine/tyrosine) phosphatases (DS-MKPs), which represent the best-characterized class [13]. Currently, at least ten main DS-MKPs have been identified [14]. Based on their subcellular localization, DS-MKPs are divided into type I, II and III. MKPs type I are located in the nucleus, MKPs type II are located in the cytoplasm, and MKPs type III are localized in both compartments [15]. The first group includes MKPs encoded by DUSP1 (MKP-1), DUSP2 (PAC-1), DUSP4 (MKP-2) and DUSP5 (hVH3). The second group includes three MKPs encoded by DUSP6 (MKP-3), DUSP7 (MKP-X) and DUSP9 (MKP-4). The last group includes three MKPs encoded by DUSP8 (hVH5), DUSP10 (MKP-5) and DUSP16 (MKP-7), which are JNK and p38 phosphatases. DUSP8 is expressed mainly in the brain, as well as in the heart and lung [16]. The different subcellular localization of DS-MKPs could be regulated by MAPK activities. In addition, DS-MKPs show differences also in tissue-specific expression, post-translational modifications and target specificity [15]. However, little is known about those phosphatases that specifically target MAP3K or MAP2K enzymes [17], which can strongly modulate the MAPKs signaling and therefore are of great interest.

## 2. The JNK Family

JNKs are a family of stress-activated serine threonine protein kinases belonging to the MAPKs. JNKs differ from classical MAPKs, such as ERK, because their activities are more potently induced in response to cellular stress rather than to mitogens [18]. There are three genes, namely *Mapk8*, *Mapk9* and *Mapk10*, encoding for ten different JNK splicing variants. JNKs regulate the activity of several transcription factors, namely c-Jun, ATF-2, Elk-1, p53, and c-Myc, as well as other factors, such as members of the Bcl-2 family [19]. They are involved in the regulation of many cellular activities, from proliferation to cell death. While JNK1 and JNK2 are widely expressed in all body tissues, *JNK3* is expressed only in the central nervous system (CNS) (with a very high level), cardiac smooth muscle and testis (low level).

*JNK1*, often with *JNK2*, has been reported to play a key role in the development of obesity-induced insulin resistance [20]. *JNK2* has also showed a key role in autoimmune disorders, such as rheumatoid arthritis [21,22], asthma [23] and cancer [24,25], and in the regulation of cancer cell survival [26].

Instead, *JNK3* is mainly involved in neurodegenerative processes like Alzheimer’s disease (AD) [27,28], Parkinson’s disease (PD) [29], cerebral ischemia [30] and other CNS disorders [31,32].

More in general, JNKs are activated in response to cytokines, environmental stresses such as heat shock and ionizing radiation, growth factors and also from internal stressors such as oxidant stress and DNA damage, DNA and protein synthesis inhibition [33] and in response to endoplasmic reticulum stress [34]. The JNK cascade is organized as the classic module of 3-MAPK. In more detail, MAP3Ks, as ASKs (apoptosis signal-regulating kinases), DLK (dual leucine zipper kinase), MEKKs (MAPK/extracellular-signal-regulated kinase kinase kinases), MLKs (mixed lineage protein kinases), TAKs (transforming-growth-factor-β-activated kinases) and Tpl2 (tumor progression locus 2) [35] interact with MAP2K (MKK4 and MKK7) that, in turn, phosphorylates JNK.

These MAPKs interact among them by protein–protein interactions mediated by different cellular-domains. It is clear that the sharing of common MAP3Ks among ERK, p38 and JNK pathways facilitates crosstalk and signal integration among MAPKs. In this context, the scaffold proteins play an important role in accelerating and isolating a single MAPK-module, preventing crosstalk with other pathways and promoting signal specificity. For example, the JNK-interacting proteins (JIP-1) scaffold protein assembles MAP3K, MKK7 and JNK, promoting the localization of signaling molecules at specific sites/compartments and coordinating cascade, preventing crosstalk. Maximum activation of JNKs is reached with the double phosphorylation of both threonine (Thr183) and tyrosine (Tyr185) residues [36]. MKK4 and MKK7 can phosphorylate both Tyr185 and Thr183; however, MKK4 has a preference for phosphorylating the Tyr residue on JNK and MKK7 for the Thr residue [9].

In this context, there are two coexisting types of MAPK/JNK cascade activation modules. The first is the simplest, where the MAPK cascade members interact by direct protein–protein interactions [37]; the second module is assembled by the scaffold protein that links together three different MAP kinases. In the latter case, the scaffold has the function of approaching the members of the signaling cascade, bringing them close together and thus accelerating the reaction cascade. In the living cell, these two types of cascade-modules are probably coexisting, and we can speculate that when it comes to the module assembled through single protein–protein interactions, the synergic phosphorylation by MKK4 and MKK7 of JNK is possible and more feasible, while in the module assembled through the scaffold, the reaction is faster, but JNK may be only once phosphorylated once, by either MKK4 or MKK7. However, the whole matter is complex and its interpretation is difficult, also due to scarce literature on the subject matter.

Concerning JNK3, while phosphorylation of Thr by MKK7 results in significant activation of JNK3, phosphorylation of Thr by MKK4 alone triggers only low levels of JNK3 activation in vitro; however, MKK4 does increase the activity of JNK that has already undergone Thr phosphorylation [8]. In addition, JNK3 has several scaffold proteins, which have different affinities to JNK3 itself and to the other MAP3K and MAP2K. To further complicate the scenario, scaffold proteins can also undergo dimerization.

Once activated, JNK phosphorylates close to 100 different protein substrates. This large number of substrates explains how the JNK signaling could control many different processes. Importantly, JNK pathway regulation also passes via its inactivation/dephosphorylation; in fact, it is inactivated, in a negative feedback loop, by MKPs.

Among the many different JNK targets, c-Jun is the elective one. This is the most studied transcription factor that, by interacting with c-Fos, forms the activator protein-1 (AP-1) complex and is involved in numerous cell activities: proliferation, apoptosis, survival, tumorigenesis and tissue morphogenesis.

While it is known that intense activation of c-Jun leads to cell death, this protein is essential for efficient cell cycle progression in physiological conditions as well [38]. In fact, in fibroblasts it was demonstrated that JNK1 and JNK2 differently regulate c-Jun phosphorylation and consequently their proliferation; JNK1 contributes more to c-Jun phosphorylation compared to JNK2, positively regulating cell proliferation, while JNK2 mostly binds to c-Jun without phosphorylating it, but instead targeting it for degradation [39]. In Schwann cells (SC), it was also shown that the overexpression of c-Jun upregulates the expression of numerous neurotropic factors that promote proliferation and migration of SC, aiding nerve repair [40]. Regarding the CNS, it was proved that the activation of c-Jun mediated by JNK1 is critical for regeneration after antiretroviral-induced peripheral neuropathy, while the activation of JNK3 leads to the onset of neuropathic pain [41]. In cerebellar neurons the activation of c-Jun induced by stress stimuli is mediated by JNK2 and JNK3, but not JNK1, leading to cell death [42]. In NGF deprived neurons, c-Jun phosphorylated by JNK3 causes apoptosis, but not oxidative stress [43]. In oligodendrocytes, while JNK3 activation, triggered by NGF, leads to c-Jun phosphorylation and cell death, in PC12 cells, the activation of c-Jun by JNK3, under the same conditions, promotes differentiation [44].

## 3. The JNK3 Isoform

The three JNK isoforms (JNK1, JNK2 and JNK3) are characterized by the same structure; in fact, JNK3 shares 92% and 87% amino acid identity, respectively, with JNK1 and JNK2. However, there are two clusters of divergent regions, identified from the amino acid sequence alignment, that are located next to each other on the protein surface in the *C*-terminal lobe of JNK3 [45]. The location of this non-conserved region suggests an extended substrate-binding site in JNK3 that may be important for substrate-binding specificity. Unfortunately, no crystal structures describing the full structure of JNK3 are currently available; the first *N*-terminal and the last *C*-terminal residues are missing in the available crystal coordinates [46]. However, JNKs show a different specificity towards their scaffold proteins. For example, all three isoforms are able to bind JIPs, but only JNK3 (not JNK1 and JNK2) can bind β-arrestin-2. This substrate specificity revealed a difference in the structure of JNK3, if compared to JNK1 and JNK2. By studying the protein–protein interactions, it was possible to identify that JNK3 has a non-conserved *N*-terminal domain, which contains nine essential amino acids for the bond to β-arrestin-2 [47].

JNK3 is primarily localized in CNS neurons and is also the most responsive isoform to stress-stimuli. In addition, it has been implicated in several neurodegenerative diseases, including Alzheimer’s disease, Parkinson’s disease and stroke. All this contributes to make JNK3 an attractive CNS drug target. Although it is well known that the JNK3 isoform is the most responsive to stress stimuli, the mechanism that regulates this responsiveness has not been determined yet. The very high homology between the three isoforms, their overlapping targets and the lack of a good specific antibody against activated JNK3 preclude better definition of this important issue.

## 4. The JNK Scaffold Proteins

Scaffold proteins, whose role is to control the activity of cellular processes driven by receptors, enzymes and channels, coordinate many different intracellular cell-signaling pathways. Scaffold proteins themselves lack of intrinsic catalytic activity but are endowed with other capacities which influence the activity of the bound enzymes. In fact, the scaffolds perform three basic functions: (1) increase the efficiency of information transfer between successive enzymes in a signaling cascade; (2) enhance the signal by reducing crosstalk between parallel cascades; (3) target effectors to specific subcellular locations [48]. In fact, the spatial and temporal organization of kinases within a cell is done by scaffold proteins that play a crucial role in regulating different cellular functions in different cellular districts.

The scaffold protein assembles a specific triad of MAP3K, MAP2K and JNK, providing a physical conduit for signal transduction assembly. This organization originates a “functional signaling module” that facilitates the signal transduction amplifying different signals thanks to a “conveyor belt” mechanism [49,50]. In addition, scaffold proteins themselves can be phosphorylated and dephosphorylated [51].

The scaffold proteins JIP and β-arrestin-2 are two important regulators of JNK signaling [52]. This signaling regulation process is even more important in polarized cells, such as neurons. The activation of JNK in the nucleus or in the postsynaptic elements produces very different response in neurons. In particular, JNK3 activation in the nucleus induces c-Jun phosphorylation, thus activating the neuronal-death program, while in the postsynaptic element, the phosphorylation of PSD-95 by JNK3 leads to down-regulation of AMPA and NMDA receptors and the degradation of PSD-95 itself, negatively regulating synaptic plasticity.

## 5. The Role of JIP Scaffold Proteins

JNK interacting proteins (JIPs) are a family of scaffold proteins encoded by four genes [53] and composed of JIP-1, JIP-2, JSAP/JIP-3 and JIP/JLP. JIP-1 exists in two different isoforms, JIP-1a and JIP-1b, both containing the JNK binding domain (the sequence able to bind JNKs), as well as JIP-2 (existing also as a splicing variant called IB2). JIP-3, also called JSAP, is structurally unrelated to JIP-1 and JIP-2 and exists in four splicing variants (JSAP-1a, b, c, d) with the highest expression levels in the brain [36]. Lastly, JIP-4 (two splicing variants) is structurally related to JIP-3 [54] and particularly expressed in round spermatids in testis [55].

**Figure 2:**
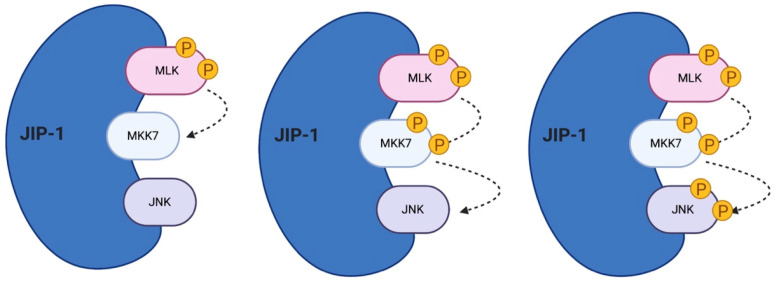
JNK’s scaffold protein JIP-1. JIP-1 is a crucial regulator of the JNK signaling pathway. It links multiple MAPK members, tethering them together and accelerating the reaction cascade. JIP-1 regulates signal transduction and helps localize pathway in components of the cell such as the plasma membrane, the cytoplasm, the nucleus, the Golgi and in neurons in the pre- and post-synaptic elements.

All the mammalian JIPs proteins are highly expressed in brain [53,56] and regulate JNK signaling during many cellular responses (Figure 2).

JIPs coordinate the JNK-module and can also bind other proteins, including kinesin light chain (KLC) [57] and APP [58], influencing the cellular response to distinct stimuli, such as cytokines (IL1, IL6), UV, oxidative stress, ischemia, NMDA stimulation and Toll-like receptor 4, and regulating distinct and overlapping functions [53]. JIP-proteins link both positive and negative JNK signaling regulators by activating, enhancing and accelerating the JNK phosphorylation/activation but also by mediating JNK dephosphorylation/inactivation [53].

More in detail, JIP-1 specifically binds all JNK isoforms and other actors of the JNK signaling cascade (MKK7, phosphatases, MEKK3, MLK3 and DLK), while JIP-2 interacts with JNK1, JNK2, MKK7, MLK2, MLK3 and DLK. However, while JIP-1 seems to bind only JNKs family members, on the contrary JIP-2 can also interact with p38 [54]. Both scaffolds show augmented expression levels in β-cells in pancreas and in neurons where they show a specific localization in cytoplasm, axons, dendritic growth cones and synapses [36]. Between JIP-1 and JIP-2, JIP-1 shows higher affinity for JNKs compared to JIP-2, but overexpression of JIP-1, as well as JIP-2, acts as an inhibitor of JNK signaling, probably because they sequester actors of the cascade. Interestingly, both JIP-1 and JIP-2 can form homodimers but also heterodimers with each other [59].

JIP-3 shows high affinity for JNK3 [60], compared to JNK1 and JNK2, and acts as a JNK activator like JIP-1 and JIP-2, but it can also bind ERK, acting as an inhibitor. JIP-3 can also bind MKK7, MKK4, MEKK1, MLK3 and ASK1 [54]. In addition, JIP-3 itself is a JNK target, since it can be phosphorylated only by JNK and not by p38 and ERK [61].

JIP-4 binds JNKs with major affinity for JNK2 and JNK3 [54]; it can also interact with KLC1 (kinesine light chain 1) but not with MKK7, MLK3, ASK1 and ERK, and, in fact, it does not appear to enhance JNK activation [53]. On the other hand, JIP-4 binds some isoforms of p38, and, in fact, it seems more involved in p38 signaling, probably representing a natural inhibitor of p38. Lastly, JIP-4 itself is a JNK and p38 target [56].

In neurons, JIPs play important roles in development and in stress responses. JIP-1 was initially characterized as an inhibitor of JNK signaling, since its overexpression inhibits JNK phosphorylation on its elective target c-Jun preventing apoptosis [62]. In fact, the JBD (JNK-binding domain) inhibitors, as D-JNKI1, mimics the overexpression of the JBD inside the cells and, avoiding the link between JNK and its physiological targets, prevents neuronal death in cerebral ischemia [63].

JIPs are mainly found in the cytoplasm, but nuclear localization has been reported under stress conditions [53,64,65]; in neurons, they localize also in the axon growth cones [64] as well as in both pre- and post-synaptic compartments [64,66]. In neurons, which are very polarized cells, the localization of the JNK module is even more important compared to other cell types, since JNK can performs different functions in each cellular compartment. JIPs, which are able to translocate among cellular districts, are therefore very intriguing proteins that can be used as tools to modulate JNK action.

## 6. The Role of β-Arrestin-2 Scaffold Protein

Arrestin proteins are known for their dual role in G protein-coupled receptor (GPCR) signaling [67], but they actually have a multifunctional role. In fact, β-arrestins act as scaffold proteins for various components of clathrin-coated endocytosis, the clathrin and AP2 (adaptin) [68], but they are also scaffolds for multiple kinases and phosphatases involved in cell-signaling (such as the MAP kinase module) [69].

**Figure 3:**
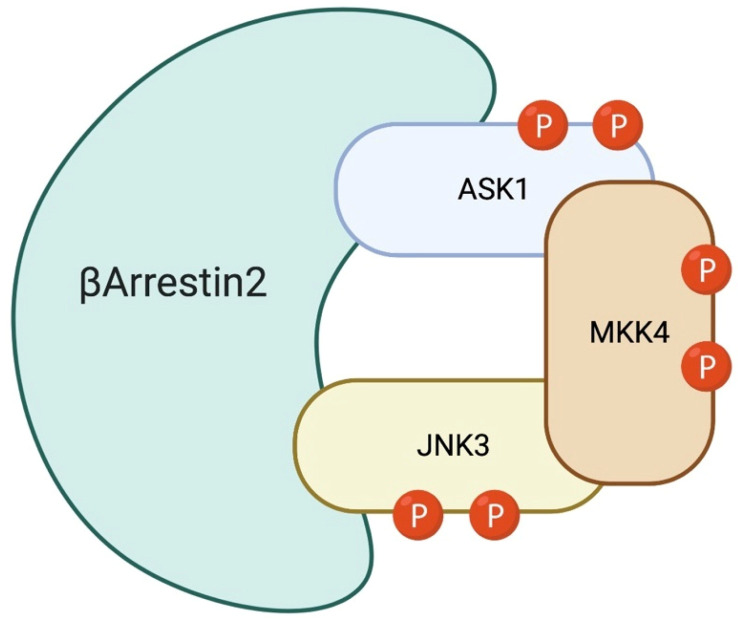
β-Arrestin-2: JNK3 scaffold. β-Arrestin-2 specifically associates with the JNK3, MKK7/4 and ASK1. Inactive-JNK3 binds β-arrestin-2 close to the MKK7/4 bond-site, facilitating full JNK3 activation. P-JNK3 loses its affinity to the scaffold and, by detaching from it, allows the binding of other inactive JNK3.

The arrestin family is composed of four members: arrestin-1, arrestin-2, arrestin-3 and arrestin-4. Arrestin-1 and -4 are also called visual-arrestins, because they are expressed in the photoreceptors of the retina, while arrestin-2 and -3 are ubiquitously expressed. Arrestin-2 is also known as β-arrestin-1, while arrestin-3 is also named β-arrestin-2. Neurons express both β-arrestin-1 and -2, but adult neurons present 10/20-fold higher levels of β-arrestin-1 in most brain regions [70,71]. Among the four different isoforms of arrestins, β-arrestin-2, in particular, specifically binds to JNK3 [72]. Recent works showed that β-arrestin-2, by changing conformation, exposes different binding domains, which link and recruit certain proteins, in particular MAPK module components. In fact, β-arrestin-2 specifically associates with the JNK3, MKK4/MKK7 and ASK1 module, and also with JNK-phosphatase, the MAP kinase phosphatase 7 (MKP7) [73]. The β-arrestin-2 scaffold can amplify different signal modules/pathways, and how this module amplifies the signal is explained by the “conveyor belt” model previously described [50]. Concerning JNK3, β-arrestin-2 binds inactive-JNK3, thus bringing it close to MKK7/MKK4, which in turn can phosphorylate JNK3 [72]. A single phosphorylation of JNK3 reduces its affinity for β-arrestin-2, while full phosphorylation leads to a higher decrease of its affinity for the scaffold, resulting in the dissociation of P-JNK3, allowing another inactive JNK3 molecule to bind β-arrestin-2 (Figure 3) [72].

In the central nervous system, β-arrestin-2 shows many additional interesting features to study; it is not only involved in JNK3 activation, but it is also involved in the fusion of synaptic vesicles (in fact it binds AP2). β-Arrestin-2 can regulate neuronal excitability, modifying CaV3 voltage dependence to suppress high-frequency action potential generation [74], and β-arrestin deletion inhibits the dendritic spine remodeling mediated by NMDA, which required active cofilin translocation to the spines in response to NMDA-receptor activation [75,76]. Furthermore, β-arrestin-2-deficient hippocampal neurons are resistant to Aβ-induced dendritic spines and synapses loss [75]. This clearly suggests an important role of β-arrestin-2 in dendritic spine plasticity/dysfunction.

The β-arrestin-2 can also act as a scaffold protein for key inflammatory signaling molecules in receptor tyrosine kinase (RTK) pathways [77], such as the NF-κB pathway [78], which proved that microglial β-arrestin-2 is a critical anti-inflammatory signal mediator [79]. In fact, β-arrestin-2 mediates the Dyn/KOR ability to limit the production of pro-inflammatory cytokines, thus inhibiting microglial inflammatory signaling and leading to a neuroprotective effect [79].

All these elements suggest an important role of β-arrestin-2 in various intracellular mechanisms linked to the plasticity/dysfunctionality of the central nervous system, indicating it as another potential key modulator in neurodegeneration/neuroprotection, as for JIP-1.

## 7. JNK3 in the Central Nervous System

The work from the Roger Davis laboratory on transgenic knockouts mice of JNK1, JNK2 and JNK3 isoforms, in which individual JNK genes were knocked out, proved that JNK1 and JNK2 are important in the development of the embryonic nervous system [80]. In fact, the single knockouts of JNK1, JNK2 or JNK3 showed no early structural abnormalities, while double knockout of JNK1 and JNK2 led to a severe dysregulation of apoptosis in the brain and proved to be embryonically lethal [81]. In addition, these studies indicate that JNK1 and JNK2 activities account for most physiological JNK actions, whereas JNK3 is the most responsive isoform to stress [30].

In particular, the Jnk3^-/-^ mice are resistant to kainic acid- and cerebral ischemia-induced neuronal death [30,82] presenting a reduced susceptibility to apoptosis induced by the AD-related protein β-amyloid in vitro [83]. Finally, Jnk3^-/-^ mice show increased dopaminergic neurons resistance after the injection of 6-hydroxydopamine [84]. Interestingly, the recent characterizations of MAPK10/JNK3 truncation mutations, in two unrelated patients, proved that the partial loss of JNK3 function induces an altered regulation of a set of post-synaptic proteins correlated with cognitive disorders [85,86]. Another case of MAPK10/JNK3 mutation was studied in a patient affected by pharmacoresistant epileptic encephalopathy. In this case, the gene was disrupted by balanced translocation, and the patient was affected by severe cognitive and motor delay, together with seizures, thus proving that JNK3 has a central role in the correct development and function of the CNS [87].

On the other hand, the suppression of one JNK isoform (knockouts/RNA-interfering) induces compensatory activation of the other isoforms, therefore complicating the clear determination of each JNK function. However, the singular functions of JNKs are probably linked to substrate specificity based on stimulus, cell type, localization inside the cell and also the temporal stimulation of the stressor. Until now, specific substrates for JNK1, JNK2, or JNK3 have yet to be determined.

Despite this, it has been established that JNK3 plays a crucial role in brain function under both normal and pathological conditions; JNK3 is involved in brain development [81], neurite formation and plasticity [88,89], in addition to memory and learning [84,90]. In pathological conditions, JNK3 has been considered as a degenerative signal transducer, and it seems to be the isoform most involved in the over-activation of JNKs after deleterious stress-stimuli in adult brain such as cerebral ischemia, TBI, hypoxia, epilepsy and many others [28].

In fact, JNK3 has been implicated in several neurodegenerative diseases, both acute and chronic. JNK3 activation has a key role in triggering apoptosis [91] and neuronal death in several neurodegenerative disorders [92,93]. In addition, recently it has been shown that JNK3 regulates “synaptopathy” [94,95,96], which is the first degenerative event of the excitatory synapses that leads to a phase of “spine dysfunction/injury” common to many brain diseases. Indeed, many neurologic and psychiatric disorders, ranging from mental retardation and autism to Alzheimer’s disease (AD), are derived by alterations in spine morphology and synapses number. Synapse dysfunction and loss has a devastating effect on neuronal communication, leading to wide ranging outcomes such as network disruption within the central neural system and muscle loss in the periphery.

Finally, the brain contains many different cell types: neurons, microglial cells, astrocytes and oligodendrocytes. To date, cell type-specific analyses of JNK signaling have not been performed; therefore, more precise and in-depth analyses are needed. This will be important to define the JNK role in these different cell types.

## 8. JNK3 is a Key Player in Synaptic Dysfunction

“Synaptopathy” is described as the first degenerative event of the excitatory synapses, which progresses to a phase of “spine dysfunction/injury” common to both neurodegenerative and neurodevelopmental brain diseases.

**Figure 4:**
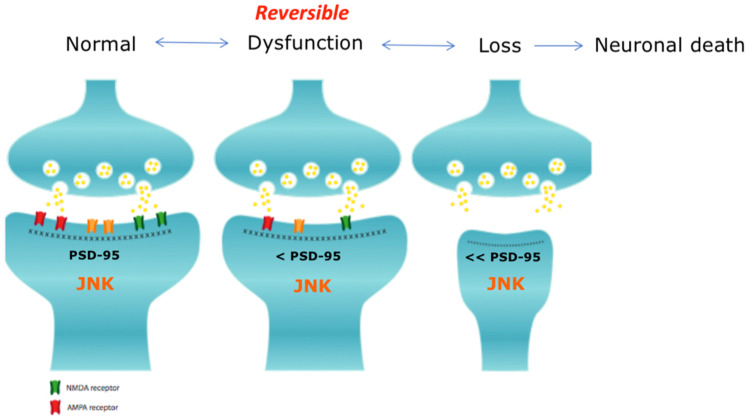
Synaptic pathology. The presynaptic and the post-synaptic elements are here represented. The first step of synaptic dysfunction is a reversible phase in which the synapse can be restored to its functional state, while the second step consists of spine loss and can progress to neuronal death [97].

Synaptic dysfunction is a dynamic process composed of an initial reversible phase, during which synaptic function is impaired, followed by spine injury that can progress to an irreversible stage, associated with synaptic loss. This second phase progresses to neuronal death and, consequently, loss of functionality of the affected brain areas and cognitive impairment (see Figure 4).

Synaptopathy can be caused by the loss-of-function of many different synaptic proteins, such as neurotransmitter receptors, proteins involved in the release of the neurotransmitters and scaffold proteins. Oftentimes the pathological processes that lead to spine injury are associated with a loss of excitatory receptors, in particular glutamate receptors [98,99]. However, PSD-95, the most abundant scaffold proteins of the dendritic spine’s post-synaptic density (PSD), plays an important role in the membrane localization of AMPA and NMDA glutamate receptors [100]. Alterations of the PSD region, such as a reduction of PSD-95, lead to the mis-localization and/or reduction of AMPA and NMDA receptors and, consequently, to the impairment of synaptic function and spine atrophy. If this impaired state persists, loss of the synapses and neuronal death will follow. In fact, reduced PSD-95 level leads to synaptic dysfunction [94,101], causing cognitive and locomotor impairments typical of many different neurological diseases, such as AD [95], Angelman syndrome (AS) [102] and Rett syndrome (RTT).

JNK is closely involved in the plasticity of the dendritic spines and in their stability [94,95,96], mainly by phosphorylating PSD-95 on the serine-295 residue, and regulates the synaptic content of PSD-95, modulating synaptic strength and contributing to plasticity [103].

## 9. JNK3 in Brain Diseases

Nowadays, it is well known that the JNK kinase is involved in a wide variety of brain diseases, in particular through its isoform JNK3. β-arrestin-2 or JIP-1 can assemble the JNK3 signaling-module, then the active-kinase can phosphorylate the transcriptional factor c-Jun, leading to caspase-3 activation and, eventually, to neuronal death. It is therefore clear that JNK3 plays a pivotal role in many different brain pathologies and injuries [92,93].

### 9.1. Ischemia

JNK3 has a key role in cerebral ischemia/reperfusion, in particular through the assembly of the Dvl-1-β-arrestin-2-JNK3 signaling module [104], and it has been proved that inhibiting JNK3 through Akt1 has a neuroprotective effect on the hippocampal CA1 pyramidal neurons after the induction of transient global brain ischemia [105,106]. Other studies have shown that the activation of the JNK3 pathway, after brain ischemia, can also occur through the assembly of an MLK3-MKK7-JNK3 signaling module, scaffolded by JIP-1; disrupting the interaction between the members of this module protects neurons against ischemic injury [107]. More in detail, pre-treatment with CoPPIX or Ros prevents the assembly of the cascade, leading to reduced JNK3 pathway activation [108], and pre-treatment with Tat-JBD attenuates the activation of JNK3 induced by ischemia and reperfusion [109]. Furthermore, JNK3 knockout neonatal mice are protected against hypoxic–ischemic injury [110].

### 9.2. Epilepsy

Different studies have also proved that JNK3 is associated with kainic acid (KA)-induced temporal lobe epilepsy; in fact, JNK3 knockout mice show decreased neuronal degeneration after KA injection [32], and pharmacological blockage of JNK3 has a neuroprotective effect against the neurotoxicity induced by KA [111], pointing to JNK3 as a key mediator of cell death during epileptogenesis.

### 9.3. Optic Neuropathies (ON)

ONs are another class of diseases in which JNK3 plays an important role. In fact, its expression and activation are upregulated in retinal ganglion cells after optic nerve axotomy, leading to retinal ganglion cell degeneration [112,113].

JNK3 activation has been observed also in other two common neurodegenerative diseases such as Parkinson’s disease (PD) and Huntington disease HD.

### 9.4. Parkinson’s Disease (PD)

PD is a neurodegenerative disease, characterized by the death of the dopaminergic neurons in the substantia nigra pars compacta. JNK3 activation has been described in many both cellular and animal models of PD. In fact, JNK3 is the main active JNKs isoform in dopaminergic neurons treated with rotenone, paraquat or 6-hydroxydopamine [114,115], and silencing or deleting the JNK3 gene reduces cell death induced by these toxins. Moreover, ablation of the JNK3 gene shows neuroprotective effects in wild-type mice treated with rotenone, paraquat or MPTP [114,115,116]. In addition, it has been demonstrated that JNK3 induces COX2 expression, a key mediator which promotes MPTP-induced toxicity in doparminergic neurons [117]. In this context, β-arrestin-2 assembles the ASK1-MKK4-JNK3 signaling module in response to MPTP injection, facilitating JNK3 activation and leading to the phosphorylation of several mitochondrial proteins and nuclear factors, resulting in mitochondrial dysfunction and, eventually, cell death [118,119]. Inhibiting the ASK1-JNK3 pathway by disrupting the interaction between β-arrestin-2 and JNK3 has been revealed as a valid strategy for preventing dopaminergic neuron loss in PD [29].

### 9.5. Huntington Disease (HD)

HD is a neurodegenerative disease that is characterized by progressive cell death in the striatum. It has been shown that JNK3 activation is triggered by the presence of pathogenic huntingtin, and in an animal model of HD obtained by treating wild-type mice with 3-NP, JNK activation and c-Jun phosphorylation have also been reported [120]. On the contrary, in this model, the ablation of JNK3 (-/-) does not show neuroprotection [121]. Moreover, one of the molecular targets of JNK3 is represented by the mammalian kinesin motor domain, which is phosphorylated by the kinase at Ser176, a specific phosphorylation associated with HD. It has been demonstrated that the phosphorylated kinesin by JNK3 is not able to bind microtubules, and axonal transport is impaired [122].

### 9.6. Alzheimer’s Disease (AD)

Another neurodegenerative disease in which JNK3 plays a pivotal role is AD. In fact, JNKs have been found to be activated in AD human brains [27,123] and also at very early stages of the disease (MCI) [123]. Importantly, JNK3 was detected in the cerebrospinal fluid (CSF) of AD patients, and its increased level is statistically correlated with the rate of cognitive decline [123], indicating that JNK3 is a key player in this disease but probably can be also a biomarker in AD.

**Figure 5:**
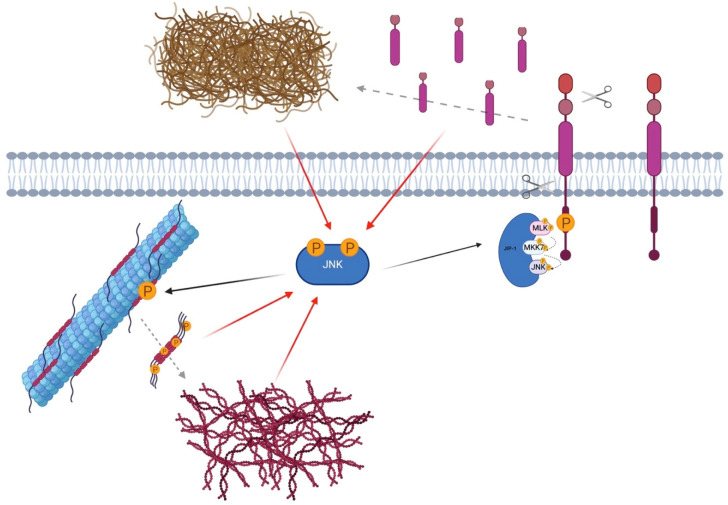
The key role of JNK in Alzheimer’s disease. Activation of JNK induces phosphorylation of the amyloid precursor protein (APP) at the Thr668 site, promoting the amyloidogenic proteolytic cleavage and the production of Aβ-toxic fragments in brain parenchyma. JIP-1, by linking the APP’s cytoplasmic tail, facilitates its phosphorylation by JNK. JNK also contributes to Tau hyper-phosphorylation and consequently to the formation of neurofibrillary tangles.

In this contest, JNK activation is well recapitulated in AD mouse models [95,96] where JNKs phosphorylate the amyloid precursor protein (APP) at the Thr668 site [124,125], inducing the amyloidogenic proteolytic processing and the production of Aβ toxic fragments in the brain parenchyma [126] (Figure 5).

Furthermore, it has been reported that JNK3 in particular is the major kinase responsible for APP phosphorylation at residue T668 (P-APP) [127]. It is known that Aβ42 can activate JNK3, which in turn phosphorylates APP at T668, facilitating the pathological amyloidogenic processing and perpetuating the Aβ42 production in a positive feedback loop [27]. In a similar fashion, JNK3 can also mediate Tau hyperphosphorylation, leading to the production of oligomeric Tau fibrils that contribute to the deposition of neurofibrillary tangles [128] (Figure 5). JNK3 is also the main JNK isoform activated in 5xFAD mice, and its activity and expression are also increased in human AD [27,129]. Moreover, Aβ42 upregulates the MLK3-MKK7-JNK3 pathway, promoting neuronal loss [130]. The importance of JNK action in the regulation of synaptic dysfunction has also been demonstrated in impairing synaptic transmission [94,101]. All this evidence leads to the idea that JNK3 might play a key role in AD, because it is directly implicated in synaptic injury and in the phosphorylation of two AD hall-markers: APP and Tau.

### 9.7. Schizophrenia

Another class of diseases in which JNK3 is involved is neuropsychiatric illness, in particular schizophrenia. In fact, it has been hypothesized that a dysregulation of multiple MAPK signaling events might be at the base of schizophrenia. JNK, together with AKT, was found to be the most dysregulated kinase in the anterior cingulated cortex (ACC) of schizophrenia patients [131]. Analysis of frontal cortical areas from schizophrenia patients showed decreased expression of JNK1, JNK2 and PSD95 and reduced phosphorylation in the ACC, indicating impaired excitatory neurotransmission [132]. Another study conducted on a relatively homogenous population in China found an association between mutations of the MAPK10 gene encoding JNK3 and schizophrenia [133]. Furthermore, the disruption of MKK7-JNK signaling affects the maternal immune response, which is linked to the development of schizophrenia [134].

### 9.8. Amyotrophic Lateral Sclerosis (ALS)

JNK3 has also been implicated in ALS. A recent study found that motor neuron (MN) death is caused by the activation of the MAP4K4-JNK3-c-Jun signaling pathway. In fact, inhibition of MAP4K4 has a neuroprotective effect on MNs and reduces mutant SOD1 accumulation by increasing cell autophagy, probably through FoxO1 [135].

### 9.9. Spinal Muscular Atrophy (SMA)

SMA is another disease in which JNK3 plays a role; mutations of the SMN1 gene causing low levels of the SMN protein trigger JNK3 activation leading to MN degeneration. JNK3 knockout partially rescues the phenotype of the SMA mice model and improved neuromuscular junction functionality together with muscle growth and longer lifespan [136].

### 9.10. Neurodevelopmental Disorders

Lastly, it has been reported that JNK might be involved not only in neurodegenerative diseases but also in neurodevelopmental diseases. In fact, JNK appears to have a role in the synaptic dysfunction observed in AS and RTT, two rare diseases characterized by both cognitive and locomotor impairment. Inhibition of JNK, through a specific JNK inhibitor peptide (D-JNKI1), protects against dendritic spine injury in vivo and prevents pathological symptoms in both syndromes recovering the cognitive and motor deficits of these mice model [102]. JNK3 was not specifically analyzed in these papers, but it is easy to assume that it will be the isoform more active in these diseases.

## 10. JNK3 as Therapeutic Target Against Neurodegenerative and Neurodevelopment Brain Diseases

JNK3 is the isoform selectively expressed in the brain and can be detected in the CFS of patients as well [123]. Importantly, JNK3 is activated by different stressors and is a key player in synaptic dysfunction, as previously described. The latter is a common denominator in both acute and chronic diseases, but also in neuropsychiatric and neurodevelopmental diseases.

For this reason, we believe that JNK3 is a promising and selective target to prevent many different brain diseases. In this context, a specific JNK3 inhibition will help to circumvent the potential side effects of a total-body JNK inhibition. JNKs regulate a myriad of cellular functions, some not linked to the pathophysiology of the brain but targeting only JNK3, allowing the stress response in the brain to be largely prevented, preserving the normal kinase function in the other organs/tissues.

## 11. Tuning JNK3 Pathway by Using JIP-1 and β-Arrestin-2

Scaffold/adapter proteins, by creating a modular complex, are able to interact in a very dynamic manner with all three kinases of the pathway. This type of organization guarantees the specificity in the activation of the substrates and the regulation of the reaction kinetics, leading to a spatial and temporal control of the signal [137] and importantly preventing the crosstalk among different MAPK family members.

JIP-1 and β-arrestin-2 are the most characterized scaffold proteins of JNK. In particular, JIP-1 presents a 15 amino-acids sequence, the JNK binding domain (JBD), which allows the binding between JIP-1-JNK and several targets [36,59].

The JNK tuning strategy that takes advantage of this substrate competitive mechanism is used by the cell permeable peptide D-JNKI1. D-JNKI1 is able to strongly inhibit all JNK isoforms in different cell types from pancreatic β-cells [62] to neurons [63].

Several studies in vitro, as well as in vivo, proved that JNK inhibitions with D-JNKI1 can offer an intriguing strategy against brain diseases, including excitotoxicity and cerebral ischemia [63], neuropathic pain [138], AD [139,140], SMA [141], deafness [142,143], ocular inflammation [144], liver injury [145], spinal cord injury [146] and traumatic brain injury [147,148]. The disadvantages of using a CPP, which is able to cross all the barriers (BBB) and inhibit all JNK isoforms in the whole body, are the possible side effects. In fact, it is worth noting that JNK plays key roles in physiological process as well, and the CPP spreads in all tissue types.

In the frame of brain diseases, exploiting the possibility to inhibit only JNK3 isoform could be a more effective and specific strategy.

What differentiates JNK3 from the other isoforms is the *N*-terminal 38 amino acid tail that mediates its specific interactions with targets and β-arrestin-2 [47]. It has been proved that mutation in this specific sequence can mediate the bond between JNK3 selective targets but also with JBD-depending partners [149].

Finally, there are also several chemical compounds identified with in-silico analysis and tested against AD neurodegeneration [150] and hypoxic insults [151], highlighting the potential of the specific inhibition of JNK3 in the brain.

## 12. JNK3 as Biomarker

Many neurodegenerative diseases such as AD and PD, for instance, are very challenging to diagnose due to the lack of selective and specific biomarkers. These brain pathologies progress silently for years and, when the first clinical symptoms appear, most of the neurons are already dead. Moreover, post-mortem brain histological examination is the only available tool to confirm the diagnosis of AD. The delay in the diagnosis has been cited as the main reason why so many drug candidates for treating neurodegenerative diseases failed in clinical trials [152]. Based on these considerations, specific biomarkers for synaptic dysfunction must be found in order to identify people associated with higher risk to develop AD or other neurodegenerative diseases. The potential role of JNK3 as a biomarker of synaptic dysfunction is supported by a clinical study showing that JNK3 levels in the CSF from AD patients were higher than those from healthy age-matched controls. In addition, JNK3 levels were related to the cognitive decline shown by patients. Specifically, the same study also showed that JNK3 strongly co-localizes with Aβ plaques in the frontal cortex of AD patients, and JNK3 expression was upregulated; on the other hand, no significant changes for JNK1 and JNK2 were found. Immediately catching the attention of the authors was the evidence that patients with higher levels of JNK3 in the CSF underwent a more rapid and severe cognitive decline compared to those with lower levels of this protein [123]. JNK3 has been suggested as a possible biomarker with a prognostic value for AD but also for determining the different pathological stages of PD. In an animal model of PD, treatment with rasagiline was able to reduce JNK3 levels in brain tissues only at the early stage, but not at the advanced stage of the disease [153], indicating a PD prognosis.

## 13. Concluding Remarks

JNK plays various functions in different biological mechanisms, both physiological and pathological. It is clear that the JNK3 is the key isoform in the CNS and that it can represent both a biomarker and a target for the treatment of brain diseases.

To date, further studies on the subject are required, for example to characterized JNK3 functions not only in neurons but also in glia and microglia cells. These cell types take part to important physiological and pathological reactions in the brain; it would be interesting to understand the importance of JNK3 in these different cell populations. Regarding neurons, it might be helpful to examine whether different neuron types and different brain regions have distinct levels of JNK3 activation. It would also be important to study if gender plays a role in JNK3 activation, since there are gender differences in the incidence of many neurodegenerative diseases. Another important issue is to find a way to measure JNK3 in the periphery in order to correlate its levels/activation state with synaptic dysfunction of the brain. This would be a turning point with important therapeutic developments for neurodevelopment, neurodegenerative and psychiatric diseases.
